# Determining the Uncertainty of X-Ray Absorption Measurements

**DOI:** 10.6028/jres.109.035

**Published:** 2004-10-01

**Authors:** Gary S. Wojcik

**Affiliations:** National Institute of Standards and Technology, Gaithersburg, MD 20899-8615

**Keywords:** cement paste, moisture, mortar, non-destructive testing, Poisson process, x-ray absorption

## Abstract

X-ray absorption (or more properly, x-ray attenuation) techniques have been applied to study the moisture movement in and moisture content of materials like cement paste, mortar, and wood. An increase in the number of x-ray counts with time at a location in a specimen may indicate a decrease in moisture content. The uncertainty of measurements from an x-ray absorption system, which must be known to properly interpret the data, is often assumed to be the square root of the number of counts, as in a Poisson process. No detailed studies have heretofore been conducted to determine the uncertainty of x-ray absorption measurements or the effect of averaging data on the uncertainty. In this study, the Poisson estimate was found to adequately approximate normalized root mean square errors (a measure of uncertainty) of counts for point measurements and profile measurements of water specimens. The Poisson estimate, however, was not reliable in approximating the magnitude of the uncertainty when averaging data from paste and mortar specimens. Changes in uncertainty from differing averaging procedures were well-approximated by a Poisson process. The normalized root mean square errors decreased when the x-ray source intensity, integration time, collimator size, and number of scanning repetitions increased. Uncertainties in mean paste and mortar count profiles were kept below 2 % by averaging vertical profiles at horizontal spacings of 1 mm or larger with counts per point above 4000. Maximum normalized root mean square errors did not exceed 10 % in any of the tests conducted.

## 1. Introduction

Over the past 5 years, x-ray absorption techniques have been used successfully in non-destructive tests to determine relative density and moisture contents in materials such as cement pastes, mortars, and wood. For example, Bentz and Hansen [1] and Bentz et al. [2] used profiles of point measurements from an x-ray absorption system to extract fundamental data on water movement in paste samples at early ages. These measurements showed that water always flows from a coarser pore layer to a finer one, as predicted by the Kelvin-Laplace equation [3], whether the difference in porosity is due to the water to binder mass ratio (*w/b*) or varying particle size distributions. These results were then used to add the drying process into the National Institute of Standards and Technology (NIST) CEMHYD3D cement hydration and microstructure development model [4,5].

It must be remembered, however, that the resulting measured signal combines information about the physical structure of the specimen and the random noise from the instrument used to do the sampling. To properly interpret the resulting signal, therefore, it is important to understand the uncertainty of the measurements. It is often assumed that the x-ray photon counting used in x-ray absorption is approximately a Poisson process in which the uncertainty is the square root of the number of counts [6,7]. This uncertainty estimate accounts for the random noise expected for a point measurement. No published data confirming this assumption for x-ray absorption measurements have been found.

Moreover, if information about the mean physical features of a specimen is desired, a point measurement or one profile of point measurements may not be representative of the larger specimen, in which case the preferred methodology would include averaging several points or profiles together. In the case of an x-ray absorption system, the proper averaging procedure may vary depending on the materials being sampled, the specimen size, the sampling time period, and the intensity of the x-ray beam. Such information is not currently available for x-ray absorption systems.

In this paper, the discussion focuses on experiments that were designed 1) to determine the uncertainty of x-ray absorption measurements and evaluate the utility of the Poisson estimate and 2) to determine how to properly average data to get a representative view of the specimen's mean relative density and composition variations. The materials considered in this study are water, epoxy, cement paste, and mortar (Table 1). The results discussed within provide an overview of the basic uncertainty of an x-ray absorption system for different machine settings and provide information about the reduction of random noise in the measurements by averaging data.

Technically speaking, what is commonly referred to as x-ray absorption is more correctly called x-ray *attenuation*, which includes photoelectric absorption, incoherent (Compton) scattering, pair production, coherent (Thomson, Rayleigh) scattering, and photo-disintegration [8]. Of these, photoelectric absorption, Compton scattering, and pair production generally dominate the attenuation of the x-ray beam. Photoelectric absorption occurs when a photon interacts with an atom and the photon energy is completely transferred to an orbital electron, which is then ejected. The process by which the x-ray photon ejects an electron from an atom and an x-ray photon of lower energy is emitted from the atom is called Compton scattering and is important for materials with low atomic numbers. For pair production, an electron and positron are created with the annihilation of the x-ray photon. This process can be important for specimens with high atomic numbers. In this paper, the term “x-ray absorption” is used, as is commonly done, to describe the integrated effect of all the processes that attenuate the x-ray beam.

In x-ray absorption systems, x rays are produced by bombarding a metal target (e.g., tungsten) with electrons that are produced by heating a metal filament (e.g., tungsten). The x-ray beam is directed toward a specimen at a selected point. The amount of energy that is transmitted through the specimen is described by Beer’s Law (e.g.,[9]):

$$\frac{I}{I_{0}}=\exp\left[-\left(\frac{\mu }{\rho }\right)\rho t\right]$$ (1)

where *I* is the x-ray intensity leaving the specimen; *I*_0_ is the x-ray intensity of the beam entering the specimen; *ρ* is the specimen density; *t* is the specimen thickness; and *µ*/*ρ* is the mass absorption coefficient. For a specimen containing several materials, the effective *µ*/*ρ* is determined by summing the *µ*/*ρ* for each material multiplied by its mass. Because each attenuating process described above is dependent on atomic number, the amount of energy transmitted will depend not only on the specimen thickness but also on the specimen composition. In Eq. (1), this dependence on atomic number is accounted for in the mass absorption coefficient.

The x-ray energy that is transmitted through a specimen reaches a detector crystal that may be composed of NaI or a combination of cadmium, zinc, and tellurium (CZT). Software then processes the signal and determines how much energy has been transmitted and outputs this as x-ray “counts.” The number of counts indicates the number of x-ray photons that have been collected by the detector. Because the amount of energy transmitted depends on specimen composition, the number of counts alone cannot be used reliably to relate the density of one specimen to that of another of equal thickness but with a different composition. However, for a given specimen, an increase in counts with time, or a positive “count difference” suggests that the specimen has become less dense. In the case of pastes and mortars after set has occurred, temporal count differences indicate changes due to the movement of water.

Work by Bentz et al. [10] with an x-ray absorption system ascertained the effects of shrinkage-reducing admixtures on self-desiccation of cement-based materials at early ages. Admixtures were found to accelerate the drying of bulk solutions while slowing the drying rate from cement paste specimens. Based on the change of counts with time (count differences), Bentz [11] concluded that cement pastes that contain fly ash lost more water and exhibited deeper drying fronts than those without fly ash. Hu and Stroeven [12] detected a complex internal moisture gradient within paste and mortar specimens with obvious top-down drying occurring only over a small zone near the specimen surfaces. Finally, Lura et al. [13] showed with x-ray absorption measurements that water transport from saturated lightweight aggregates to hydrating cement paste occurred over a distance of at least 4 mm.

The data in these previous studies show some scatter and without an estimate of the uncertainty of the measurements, it is difficult to determine if the variations seen are actual physical features of the specimen or random noise. To reduce scatter and to reveal the mean trends in the data, it may be necessary to average many data points together.

In addition, it is not clear if the results of these previous studies are applicable to larger mortar and concrete specimens for which the smaller paste and mortar specimens are intended to be a model. For example, the presence of an interfacial transition zone around aggregates and different particle packing in mortars and concrete may cause water movement in mortars and concretes that is different from that in cement pastes. Also, the mortar specimens tested by Hu and Stroeven [12] had a minimum dimension of 5 mm and a maximum aggregate size of 4 mm, violating a rule-of-thumb that to obtain a representative volume of a specimen, the ratio of the minimum specimen dimension to the maximum aggregate size should be five or larger [14]. For a mortar with a maximum aggregate size of 4 mm, the minimum horizontal specimen dimension should be at least 20 mm and for a concrete with a maximum aggregate size of 40 mm, the minimum specimen dimension should be at least 200 mm. Due to expected density and composition variations in these larger samples caused by the presence of aggregate, many points of the specimen would need to be measured and averaged to decipher the specimen’s mean structure. To properly interpret the counts and their temporal changes, it is necessary to understand the inherent uncertainty introduced into the measurements by the machine itself. Determining the best way to sample the mean features of larger specimens and determining the uncertainty of an x-ray absorption system’s measurements are the goals of this paper.

## 2. Methodology

### 2.1 X-Ray Absorption System

The x-ray absorption system used for this study was designed and constructed by GNI^1^ and is located at NIST. The apparatus uses a tungsten filament and a tungsten target to generate x rays and the detector contains a CZT crystal. The x-ray beam exiting the source is approximately 4.5 mm in diameter. The size of the beam entering the detector after passing through the specimen is controlled by selecting one of seven collimators, ranging in area from 0.0393 mm^2^ to 9.0 mm^2^ (Table 2). The collimator openings are circular, square, or elliptical in shape. Energy levels are detected in 256 channels. Because different materials have different absorption cross-sections, the shape of the spectra can be used to identify the presence of certain materials in a specimen. In this work, however, the total number of counts for a given spectra is used for analysis in this work, as has been common in previous studies [1,2,10–13,15]. The x-ray beam intensity is determined by setting the current (0 µA to 3000 µA) to heat the filament to produce electrons and a voltage (20 kV to 60 kV) to accelerate the electrons toward the target. The period of time over which a given point is sampled by the detector is referred to as the “integration time” and can range from 1 s to 10^4^ s. Motor movement of the x-ray source is controllable in the horizontal and vertical directions and the movement of the detector is controllable in three dimensions with a resolution of 0.1 mm. The error in the detector position in this study was less than 0.05 mm in the horizontal and vertical directions (in the plane of the specimen) and 0.3 mm in the direction perpendicular to the front and back face of the specimen. Motor movement and x-ray beam intensity are controllable with software that allows tasks to be created containing settings for all of the variables mentioned above.

### 2.2 Specimens and Measurement

**Fig. 1:**
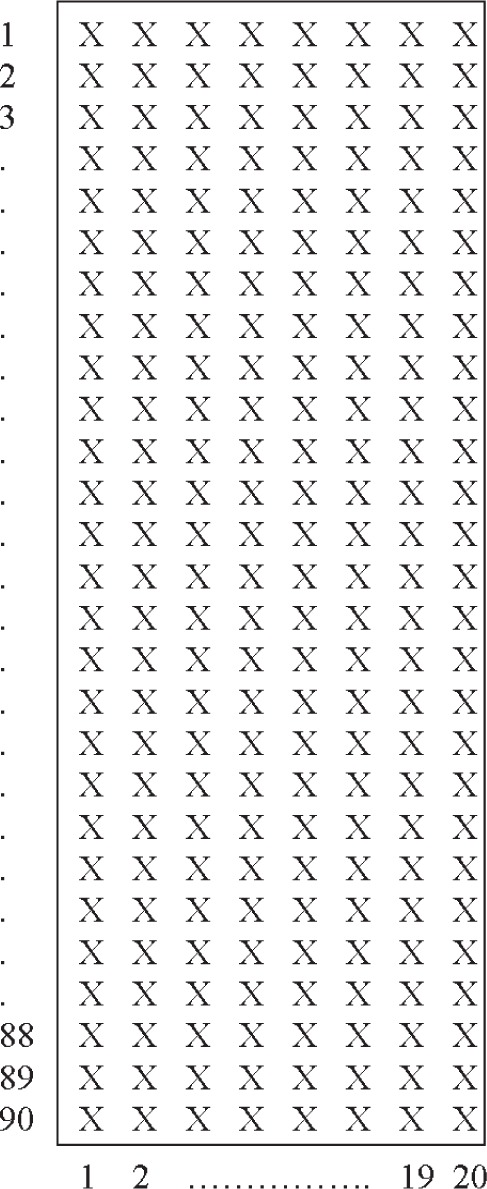
Schematic cross-section of a paste or mortar specimen showing the locations of measurement points in the vertical and in the horizontal (the x-ray beam would travel into the page). The spacing between each point in the vertical and in the horizontal is 1 mm. For each specimen for which complete scans were performed, 20 vertical scans of 90 points each were performed, starting at every 1 mm in the horizontal. To get statistics for the vertical profiles as a function of different horizontal scanning resolutions, vertical scans at selected horizontal locations were averaged together at each vertical point. For example, for a horizontal scanning resolution of 2 mm, the points at horizontal positions of 1, 3, 5, 7, 9, 11, 13, 15, 17, and 19 were used.

Many of the measurements described here were performed as the appropriate specimen size, specimen holder, and averaging procedure for mortar and paste specimens were determined. Experiments were performed on water, cement pastes, and mortars to determine the variability and uncertainty in counts as a result of changing various machine settings including the integration time, x-ray source intensity, and collimator size, and as a result of changing averaging procedures including the number of scanning repetitions and horizontal scanning resolution (Fig. 1). In preliminary tests studying the drying of pastes and mortars, a vertical resolution of 1 mm was found to provide the necessary vertical detail [15] and so a focus of this work was on count variations from changes in horizontal resolution. A test with an epoxy block in water was used to investigate the machine’s ability to decipher the proper location of the edges of an object.

The paste and mortar specimens had a water to binder mass ration (*w/b*) of 0.4 and the mortar had a binder to sand mass ratio (*b/s*) of 0.6. The binder was an ASTM Type I cement [11] and the sand was a mix of four different fine aggregate gradations with a maximum size of 2.36 mm (Table 3). The aggregate mix was designed to best approximate the Fuller curves of aggregate gradation, which produce the maximum packing density [17]. The specimens were mixed in a standard constant speed laboratory mixer with a mix volume of about 200 ml. Cement powder was added to water and aggregate (where appropriate) and mixed for 60 s at 67.5 Hz (4050 rad/s) and then at 167.3 Hz (10 040 rad/s) for 30 s. Once placed in the specimen holders, the specimens were placed on a vibrating table in a vacuum chamber to remove any large air bubbles. The specimens were sealed and cured for at least 28 d and so there were essentially no further temporal or spatial changes in microstructure during the sampling.

The specimen containers were composed of acrylic or plastic. Unlike glass or quartz, acrylic and plastic do not significantly absorb or scatter x rays. The acrylic containers were custom made out of 0.95 mm acrylic sheets and produced specimens approximately 26 mm wide, 21 mm deep, and 100 mm high. Note that the dimension described as “deep” represents the path length of the beam through the specimen or the thickness of the specimen. The larger plastic containers were approximately 20 mm wide, 55 mm deep, and 60 mm high. The smaller plastic containers were about 10 mm wide, 10 mm deep, and 80 mm high. All containers were sealed with caps and epoxy. No appreciable mass loss was noted for any of the specimens.

Because the degree of attenuation of the x-ray beam depends in part on the specimen thickness, the variation of the thickness of the specimens from top to bottom should be examined to ensure that they are uniform. Such thickness variations are the result of uneven dimensions of the specimen containers. Variations in the thickness of the specimen were less than 0.4 mm for the acrylic and the larger plastic containers from the top to the bottom. Variations for the smaller plastic containers were about 0.7 mm, with the thickest part of the specimen near the top. While the variations in thickness of specimens from the acrylic and larger plastic containers produced no noticeable influence on x-ray counts, the counts for a water-filled smaller plastic container decreased by approximately 3 % from bottom to top, consistent with greater attenuation near the top (see Sec. 3.4).

### 2.3 Uncertainty Analysis

The process of radiation counting is well-defined by a Poisson distribution for which the mean is equal to the number of counts over a given time period and the standard deviation is equal to the square root of the number of counts [6,7]. As mentioned earlier, this uncertainty measure quantifies random noise in point measurements for counting processes. The developer of the x-ray absorption apparatus, GNI, states that the Poisson standard deviation is roughly the uncertainty of the measurements. For example, a measurement of 10^4^ counts has an uncertainty of approximately 100 counts or 1 % of the total counts. Likewise, for 1000 counts, the uncertainty is 32 counts or 3 % of the total counts. In other words, fewer counts have a higher uncertainty as a percentage of the counts.

The use of the Poisson approach to estimate uncertainty is only warranted in the x-ray absorption process for one point or for multiple points when the specimen is uniform in composition and thickness. Different points across a heterogeneous specimen have different counts not due to random noise, but to real physical features. For determining the average uncertainty of a vertical scan of a heterogeneous specimen, then, statistical measures other than the Poisson approach would be more appropriate. The variability and the uncertainty of the measurements are also described in this work with the standard deviation (*SD*) and with the root mean square error (*RMSE*) and the *SD* and *RMSE* normalized by the average number of counts per specimen point for a given measurement (*NSD* and *NRMSE*):

$$NSD=\frac{1}{N}{\left[\sum {\left(x-N\right)}^{2}\right]}^{0.5}$$ (2)

$$NRMSE=\frac{1}{N}{\left[\frac{\sum {\left(x-x_{c}\right)}^{2}}{n}\right]}^{0.5}$$ (3)

where *x* is the number of counts at a given point, *x*_c_ is the number of counts at a given point averaged over several samples (the “correct” or “true” value; see below), *n* is the number of points, and *N* indicates the average number of counts per point. To compute the *RMSE* and *NRMSE*, the average of many measurements was selected as the “correct” or “true” measurement.

## 3. Results

The results for several experiments designed to test the utility of the Poisson uncertainty estimate are presented first. After this, the influence of averaging measurements on the scatter and uncertainty in the data is discussed.

### 3.1 Utility of the Poisson Uncertainty Analysis

The utility of the Poisson approach to x-ray absorption measurement uncertainty was tested by comparing the Poisson estimate to the *NRMSE* and the *NSD* of measurements for water, paste, and mortar specimens. In experiment *OP* (Table 1), one point in the water specimen was sampled 100 times at three different intensities. In hindsight, performing this same task for the paste and mortar specimen may have been enlightening, but these measurements were not done.

Because many studies using x-ray absorption have involved count profiles, vertical count profiles for the water, paste, and mortar specimens (Fig. 1) were measured four different times at a fixed horizontal location for three different intensities in experiments *WVP* and *IVP* (Table 1). For the vertical profiles, the *NSD* can only be used as a measure of uncertainty for the water specimen (or other uniform specimen). As will be shown below, the *NSD* for the paste and mortar specimens provides information about the density structure and composition of these specimens that is not measurement error.

As the x-ray intensity increased, the number of counts from the water specimen point sampled 100 times increased (Table 4). All of the uncertainty indices decreased as the number of counts increased. For example, the Poisson estimate of uncertainty decreased accordingly from 2.7 % at intensity A to 0.3 % at intensity C while the *NSD* decreased from 3.6 % to 0.5 % over the intensity range. When sampling one point repeatedly, the Poisson estimate slightly underestimated the uncertainties estimated by the *NRMSE* and the *NSD*. Small variations in the thickness of the specimen or the container housing it may increase the uncertainty estimates from the *NRMSE* and *NSD* over what might be expected from the random noise indicated by the Poisson estimate. In general, the Poisson estimate provides a reasonable approximation to the uncertainty of point estimates.

The uncertainties of the vertical profiles from the water, paste, and mortar specimens (experiment *IVP*; Table 1) also decreased as the x-ray source intensity increased (Table 5). In these experiments, four vertical profiles at a fixed horizontal location were measured in each specimen. The values for *NSD* and *NRMSE* in Table 5 were computed in two ways, with the average profile at the highest intensity being used as the true value for the *NRMSE* calculation. First, the *NRMSE* and *NSD* of each individual profile for each intensity were averaged together to determine mean values (*NRMSE*a and *NSD*a in Table 5). Second, an average vertical profile for each intensity was determined and then compared to the true profile (*NRMS*b and *NSD*b in Table 5). These two methods were used to demonstrate how constructing a mean profile from several vertical profiles over the same points can reduce the uncertainty. Strictly speaking, the use of the Poisson estimate for several different points of heterogeneous specimens like pastes and mortars is not warranted. The Poisson values, however, were computed to examine how the Poisson estimate would perform with simple vertical profiles.

**Fig. 2:**
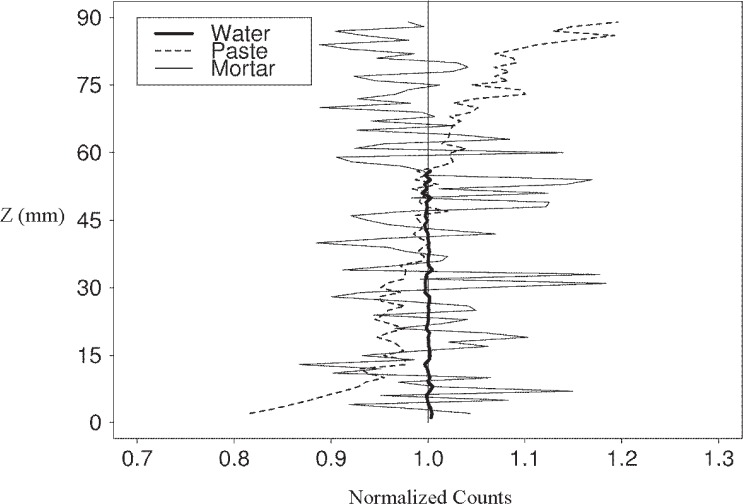
Vertical profiles of normalized counts for water, paste, and mortar specimens from experiment *IVP*. Each mean profile was determined by averaging together four vertical scans at one horizontal location.

For the water specimen, creating a mean profile reduced the *NRMSE* and the *NSD* by a factor of two, as might be expected for a Poisson process. For intensity B, *NRMSE*a is 1.2 % and for *NRMSE*b is 0.6 % (Table 5). The *NSD*a is 1.2 % and the *NSD*b is 0.5 %. In fact, the averaging reduced the *NRMSE*s and *NSD*s below the Poisson uncertainty estimate (0.8 %). The reduction in the paste’s *NRMSE* is 45 % and in the *NSD* is less than 20 %. For the mortar, the *NRMSE* and *NSD* are reduced by 33 % and 2 % or less, respectively. The reason that the *NRMSE* is reduced more by averaging than the *NSD* for the paste and mortar is that random noise is averaged out with the *NRMSE* calculation while the *NSD* is indicating some physical density or composition variations in the specimens (Fig. 2). The *NRMSE* and *NSD* of the water specimen are reduced approximately the same amount as might be expected for a uniform specimen. Moreover, the *NSD* and *NRMSE* of a uniform specimen should approach zero when the random noise is averaged out, as is the case with the water specimen.

This comparison of *NRMSE* and *NSD* may be a convenient way with which to distinguish between random noise from the measurements and physical detail in the specimen (Table 5; Fig. 2). At higher counts, the *NRMSE* and *NSD* for the paste and mortars are considerably different. The *NRMSE* of the mortar at intensity *C* approaches zero (1.5 %), as random noise is averaged out of the measurements. At the same time, *NSD* converges to a high variability (8.3 %), indicating more detail in the profile.

The Poisson estimates of uncertainty for each of the specimens are within 25 % to 60 % of the *NRMSE* and *NSD* (for the water specimen) estimates, depending on the averaging procedure that is used. While the magnitudes may differ, the trend in uncertainties (a reduction by a factor of two when the number of counts increases by a factor of four by averaging four water profiles together) is indicative of a Poisson process. At the highest intensities and for these simple experiments, the uncertainties are less than 2 % and the Poisson estimate provides a reasonable measure of the uncertainty. The utility of the Poisson approach in more complex experiments will be discussed in Sec. 3.3.

### 3.2 Influence of Machine Settings: Integration Time and Collimator Size

As seen in the previous section, increasing the x-ray intensity and the number of counts reduces the uncertainty of point measurements and vertical profiles, in general agreement with a Poisson process. Increasing the integration time and the collimator size at a fixed x-ray intensity will also increase the number of counts. In this section, the effect of changing the integration time and the collimator size on the uncertainty is explored. With the integration time experiments, only a water specimen was used while for the collimator experiment, water, paste, and mortar specimens were examined.

#### 3.2.1 Influence of Integration Time (Experiment *WVP*)

**Fig. 3:**
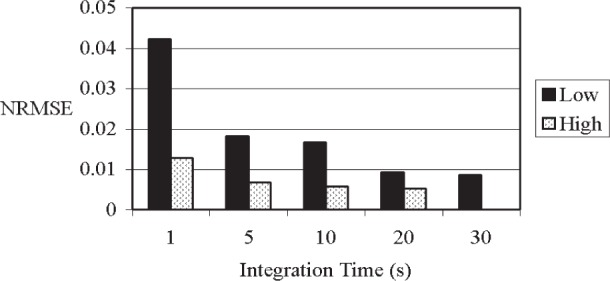
Normalized Root Mean Square Errors (*NRMSE*) as a function of integration time for experiment *WVP* at low (30 kV; 200 µA; ≈850 counts per second) and high (35 kV; 700 µA; ≈12 000 counts per second) x-ray source intensities. The data from the 30 s integration time at the high intensity was used as the true profile for the calculation of the *NRMSE*. See Table 1 for more information on experiment *WVP*.

To demonstrate the influence of integration time on the *NRMSE*, the same water specimen as used above was scanned vertically at a high intensity (35 kV, 700 µA) and at a low intensity (30 kV, 200 µA) for integration times of (1, 5, 10, 20, and 30) s. After five seconds of integration, x-ray counts were ≈4300 and ≈59 600 for the low and high intensities, respectively. The 30 s scan at the high intensity was assumed to be the true value for the computation of *NRMSE*s. As the integration time increased, the *NRMSE*s decreased (Fig. 3). For example, at the high intensity, the *NRMSE* decreased from 1.3 % to 0.5 % when the integration time was increased from 1 s to 20 s. At the high intensity, integration times of 5 s and higher result in practically the same *NRMSE*. The *NRMSE*s are again slightly higher than the uncertainty estimated from the square root of the number of counts. In general, integration times of 5 s or longer produced *NRMSE*s that are less than 2 % for both intensities tested. Note that the high intensity produced *NRMSE*s that are two to three times lower than those produced by the low intensity. Higher intensities and longer integration periods, then, decrease the uncertainty in the measurements, as expected for a Poisson process.

#### 3.2.2 Influence of Collimator Size (Experiment *CVP*)

##### 3.2.2.1 Experiment *CVP* With Water

**Fig. 4:**
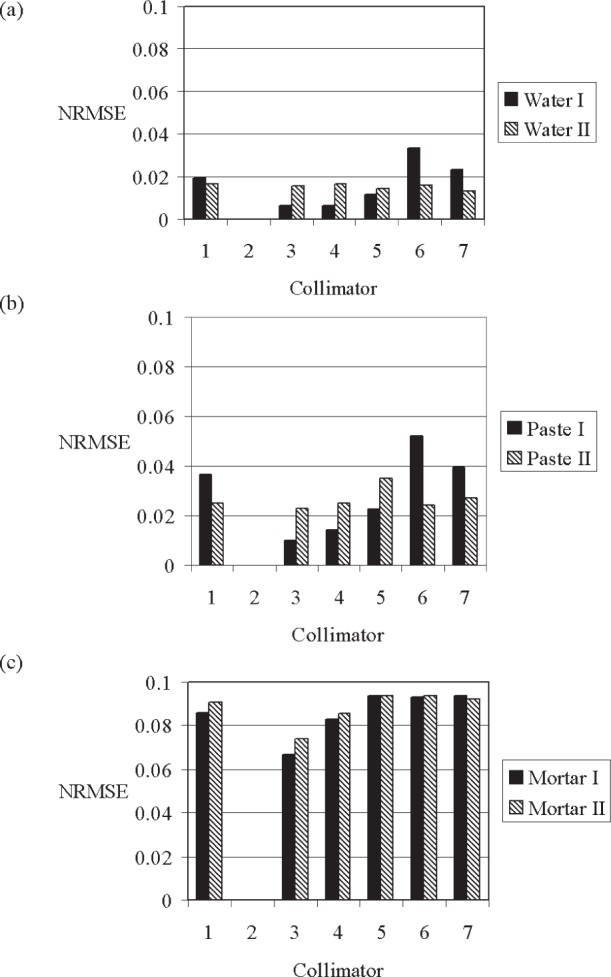
Normalized Root Mean Square Errors (*NRMSE*) for each collimator (experiment *CVP*). (a) Water. (b) Paste. (c) Mortar. See Table 1 for more details about experiment *CVP* and see Table 2 for more information about the collimators. The “I” in the legend label refers to scans in which the x-ray source intensity was kept constant (allowing counts to vary by collimator size) while “II” refers to scans in which the counts were held constant. For Water II, the average counts were within ±0.6 % of 17 170 counts. For Paste II, the average counts were within ±1.8 % of 17 192 counts. For Mortar II, the average counts were within ±2 % of 17 637 counts.

At a given x-ray source intensity, the number of counts changes proportionally with the change in collimator size (see “Water” data in Table 2). For vertical scans of the water specimen discussed above, at a constant x-ray source intensity, the *NRMSE*s of the measurements from all of the collimators referenced to collimator #2 (the largest opening area) increased as the collimator size decreased (“Water I” in Fig. 4a). This dependence on collimator size is directly related to the number of counts measured for each collimator. To demonstrate this fact, the *NRMSE*s from scans for each collimator at a constant intensity (Water I) were compared with those from scans in which the counts were held relatively constant (“Water II” in Fig. 4a). As the collimator opening gets smaller, the counts in Water I decreased and the *NRMSE*s increased, as mentioned above. When the x-ray counts for each collimator are kept at ≈17 000 counts per point by adjusting the x-ray source intensity for each collimator (Water II), the trend in *NRMSE*s seen in Water I is no longer present. Therefore, for a specimen that is uniform in composition and density, increasing the collimator size reduces the uncertainty because the number of counts is increased for a given integration time.

##### 3.2.2.2 Experiment *CVP* With Paste and Mortar

For the paste specimen, the *NRMSE*s vary inversely with the collimator size when using a constant x-ray source intensity. As the collimator size decreases from collimator #3 to collimator #6, the *NRMSE*s increase from 1 % to 5 % (Fig. 4b). It is of interest to determine whether this variation in *NRMSE*s indicates that greater physical detail has been revealed (*NRMSE*s increase as the collimator size decreases) or that random noise due to x-ray beam intensity fluctuations or machine positioning errors from the measurements with the smaller collimators is greater (smaller collimators have fewer counts and so would have higher *NRMSE*s as discussed earlier). Tests were performed in which the counts were kept constant for each collimator by adjusting the x-ray source intensity, thereby eliminating the effect of counts on the *NRMSE*s. There is some indication that the *NRMSE*s for all collimators with constant counts follow the same pattern as with the variable counts, suggesting that the increase in paste *NRMSE*s for smaller collimators indicates greater detail in the profiles (the data for collimator #6 are the exception). However, the data are not conclusive.

Another way to determine whether or not increased *NRMSE*s indicate increased detail or are the result of random noise is to compute *NRMSE*s and *NSD*s for a mean profile from several vertical scans. Machine positioning error would be averaged out when a sufficient number of scans were averaged together (assuming the errors are random) and the *NRMSE*s would approach zero. In this case, the *NSD* would indicate the amount of detail in the profile. Only one vertical profile was measured for the *CVP* experiments and so it is not possible to evaluate these statements for the *CVP* experiments at this time.

For the mortar specimen when using a constant x-ray source intensity, the largest collimators also result in the lowest *NRMSE* (Fig. 4c). However, the values for the smallest collimators (collimators #1, #5–7) are similar, in contrast to what was found for the paste and water specimens. This pattern for the mortar also exists when the number of counts is kept constant for each collimator, suggesting that random noise is not responsible for the pattern. As the collimator size decreases, the profiles become more detailed as indicated by the larger *NRMSE*s. With a vertical resolution of 1 mm, the profile data from collimators #2 and #3 (Table 2) are smoothed as some overlapping areas occur between adjacent points. For the smaller collimators, this smoothing does not exist. Moreover, the data from the smaller collimators show more small-scale variability due to a smaller field of view and the error in the positioning of the x-ray beam/detector that combine to maintain a high *NRMSE*.

**Fig. 5:**
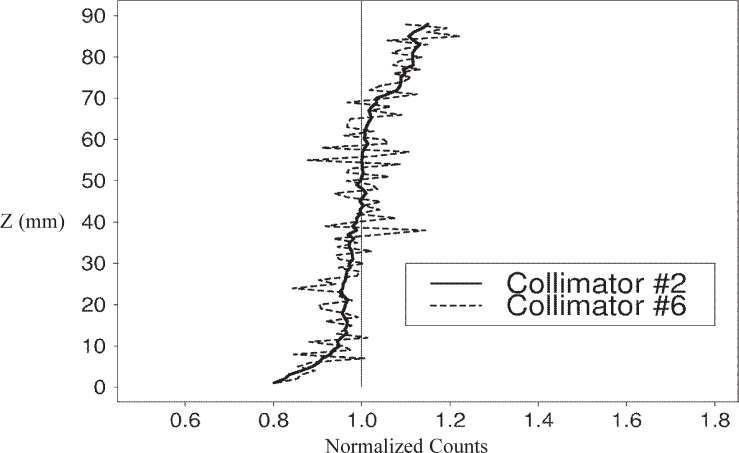
Normalized counts for the paste specimen in experiment *CVP*. The data were taken from vertical profiles with collimator #2 and collimator #6. See Table 1 for more information about experiment *CVP* and Table 2 for more information about the collimators.

**Fig. 6:**
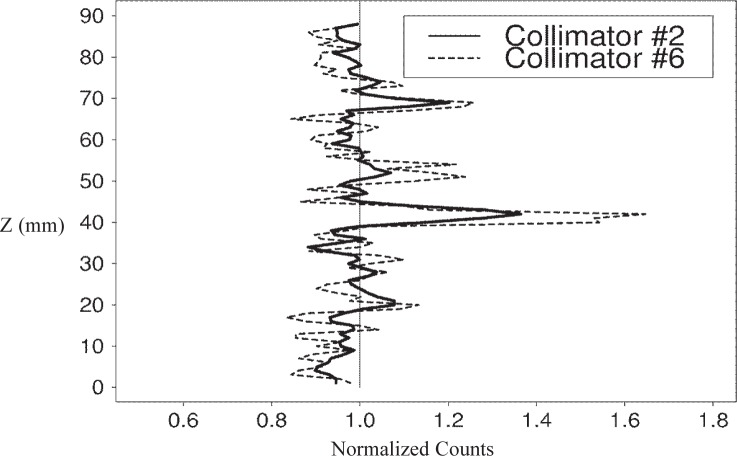
Normalized counts for the mortar specimen in experiment *CVP*. The data were taken from vertical profiles with collimator #2 and collimator #6. See Table 1 for more information about experiment CVP and Table 2 for more information about the collimators.

When comparing the vertical profiles of the specimens that showed the lowest and highest *NRMSE* from the collimator experiments, the effect of the larger collimator size on reducing variability is clear. The paste and mortar profiles for collimator #2 show much less structure than the collimator #6 profiles (Figs. 5 and 6). So with a larger collimator, it is possible to reduce the amount of small-scale variability that is seen in the profiles, perhaps elucidating larger scale features of the specimen.

The collimators are of known size and so it is possible to estimate the number of counts measured with a given collimator at a given intensity if the count data for just one of the collimators is available. The “Area Factor” is calculated by comparing the area of each collimator to the area of the largest collimator (collimator #2). For example, the Area Factor for collimator #3 is 2.9, which is determined by dividing the area of the largest collimator (9 mm^2^) by the area of collimator #3 (3.14 mm^2^; Table 2). If the counts for collimator #2 are divided by this Area Factor, an estimate of the number of counts for collimator #3 is obtained. For collimators #3-#7, the Area Factor determined from the water, paste, and mortar data is generally consistent with the machine specifications. The small differences between the expected Area Factor and the determined Area Factor perhaps result because the x-ray source intensity entering the collimator may not be uniform over the whole area due to a non-uniform x-ray source. The data for the water, paste, and mortar specimens provide an Area Factor of about 125 for collimator #1, while the machine specifications suggest that it should be 229. This data suggests that the dimensions of collimator #1 as provided are not correct and that the collimator is larger than specified.

### 3.3 Influence of Averaging Procedures With the Paste and Mortar Experiments

The water, paste, and mortar experiments discussed above showed that the uncertainty decreases as the number of counts increase, whether that increase in counts is due to increased intensity, increased integration time, or increased collimator size. These findings are consistent with those from a Poisson process. The results of the paste and mortar collimator experiments, however, suggest that the physical structure of the specimen may invalidate the use of the Poisson estimate of uncertainty for some specimens since the *NRMSE*s did not scale in the same way with the number of counts (Fig. 4).

The vertical profile results presented thus far were for vertical profiles at one horizontal location. In this section, the discussion focuses on how averaging over larger specimen domains influences the uncertainty of the estimated mean vertical structure of the specimen. Two different averaging procedures were examined: 1) averaging together vertical profiles at different horizontal locations (different horizontal resolutions; experiment *XVP* in Table 1) and 2) averaging a number of scanning repetitions (experiment *RVP* in Table 1). For these experiments, the data from the scans with a horizontal and vertical resolution of 1 mm (Fig. 1), averaged over four scans, at x-ray intensity settings of 43 kV and 700 µA (intensity *C*) was used as the true mean vertical count profile of the specimens. Three intensities were used (Table 1).

**Fig. 7:**
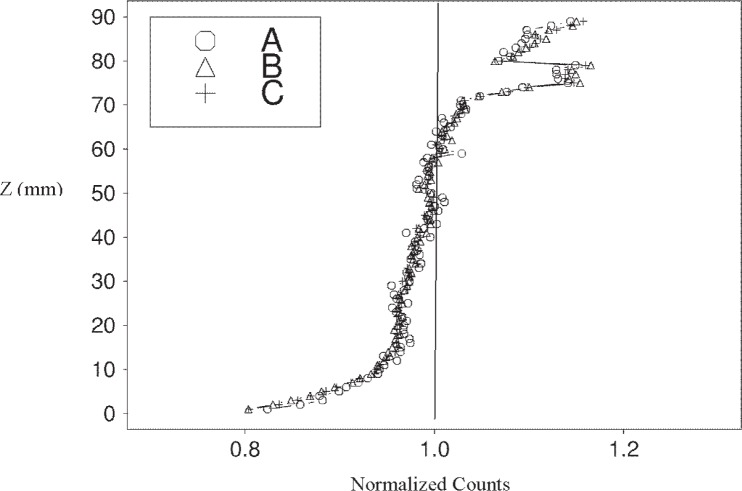
Normalized counts from the vertical scans of a paste specimen in experiment *IVP* for three different intensities (*A, B*, and *C*). See Table 1 for more details. The profiles were determined by averaging the points at each level from 20 scans.

**Fig. 8:**
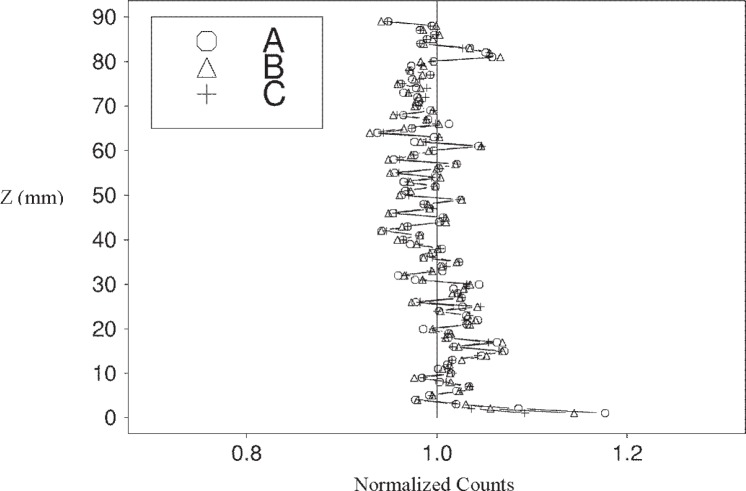
Normalized counts from the vertical scans of a mortar specimen in experiment *IVP* for three different intensities (*A, B*, and *C*). See Table 1 for more details. The profiles were determined by averaging the points at each level from 20 scans.

The paste specimen at intensity *C* has a *NSD* of roughly 7.1 %, while that for the mortar sample is about 2.9 %. The reason that the paste (Fig. 7) has a higher overall vertical variability than the mortar (Fig. 8) is that the paste counts show a distinct vertical trend with higher counts near the top of the specimen and fewer counts near the bottom. This trend is the result of more efficient compaction and possible subsequent bleeding of the paste specimen, induced by the vibrating table, than of the mortar specimen due to the smaller average particle size in the paste. The result is better packing and higher densities near the bottom of the specimen and lower density near the top, as observed previously for *w*/*b* = 0.75 pastes [1,2].

#### 3.3.1 Influence of Horizontal Measurement Spacing (Experiment *XVP*)

In this experiment, vertical profiles of counts at different horizontal locations were averaged together to create a mean profile. The goal was to determine how mean profiles created by averaging profiles from varying horizontal resolutions compare to the mean profile created by averaging profiles at the maximum horizontal resolution of 1 mm (Fig. 1). This procedure will help to determine the minimum horizontal resolution needed to approximate the overall mean profile of a specimen. Note that because vertical profiles from different portions of the specimen with differing structures are averaged together, the results below should not be considered necessarily as machine error. Instead, they indicate the utility of averaging procedures to approximate the mean profile.

**Fig. 9:**
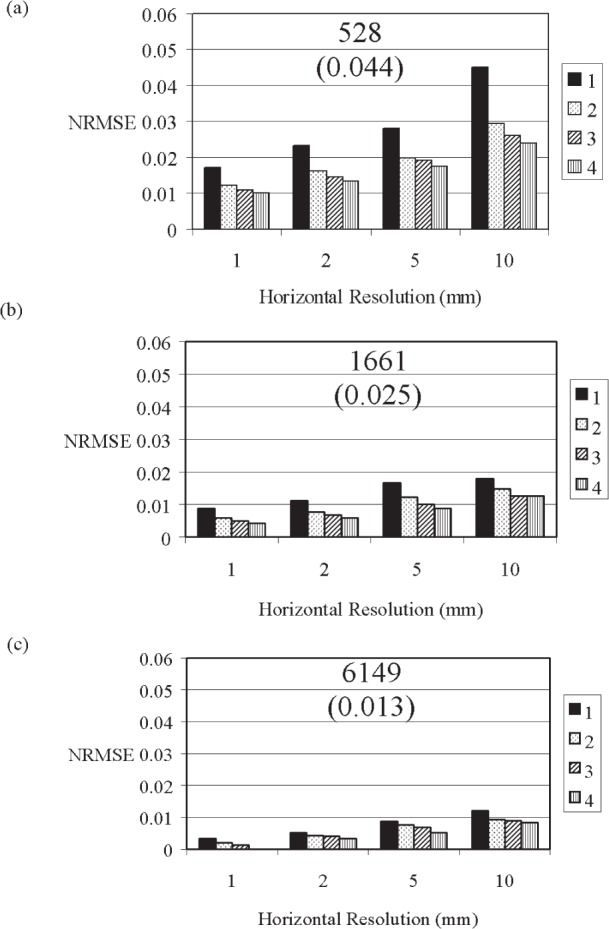
Normalized Root Mean Square Errors (*NRMSE*) for the paste specimen in experiment *IVP* as a function of horizontal scanning resolution and the number of scans performed as indicated in the legend. The true value for the calculation of *NRMSE* was assumed to be the data in which four scans of the specimen were averaged together at 1 mm horizontal resolution at intensity *C*. See Table 1 for more information. (a) Intensity *A*. (b) Intensity *B*. (c) Intensity *C*. The numbers at the top-center of each chart indicate the approximate average number of counts per point for the corresponding x-ray source intensity. The values in parentheses below the number of counts are the Poisson estimates of uncertainty or the inverse of the square root of the number of counts.

**Fig. 10:**
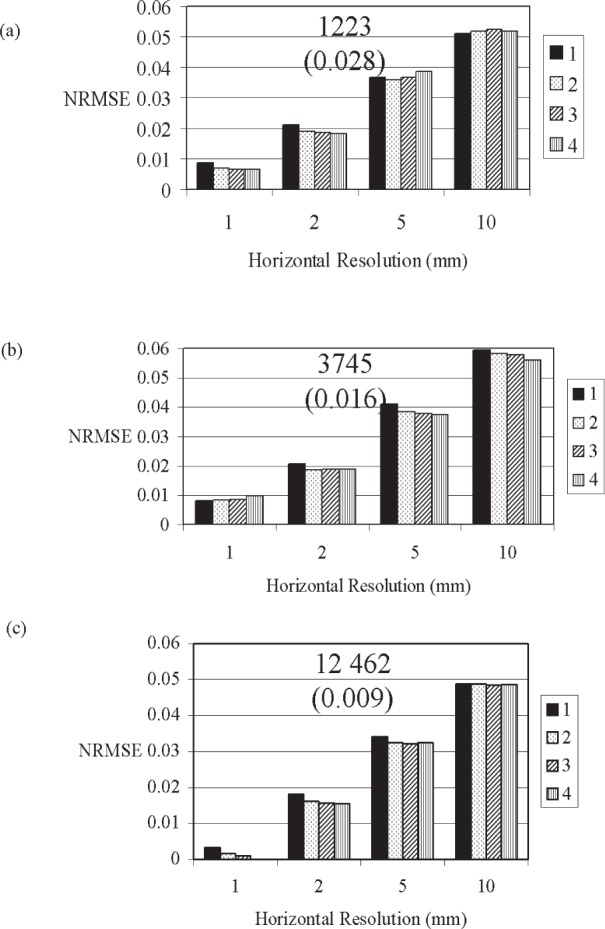
Normalized Root Mean Square Errors (*NRMSE*) for the mortar specimen in experiment *IVP* as a function of horizontal scanning resolution and the number of scans performed as indicated in the legend. The true value for the calculation of *NRMSE* was assumed to be the data in which four scans of the specimen were done at 1 mm horizontal resolution at intensity *C*. See Table 1 for more information. (a) Intensity *A*. (b) Intensity *B*. (c) Intensity *C*. The numbers at the top-center of each chart indicate the approximate average number of counts per point for the corresponding x-ray source intensity. The values in parentheses below the number of counts are the Poisson estimates of uncertainty or the inverse of the square root of the number of counts.

As the horizontal spacing between vertical profiles increased, the *NRMSE* for both the paste and mortar vertical profiles increased (Figs. 9 and 10). Moreover, the *NRMSE* of mortar scans is more dependent on the horizontal resolution than those for paste scans due to the greater heterogeneity of the mortar mixes caused by the aggregate. The *NRMSE* for the mortar scans at a horizontal resolution of 10 mm at intensity *C* is nearly 5 % while that for a 1 mm horizontal resolution is about 0.2 % (Fig. 10c). For these same conditions, the paste *NRMSE*s vary from about 1 % to 0.2 % (Fig. 9c). For the paste, a horizontal resolution of 10 mm or less at intensity *C* is sufficient to produce *NRMSE*s of less than 2 %. For the mortar, a horizontal resolution of 2 mm and counts greater than 4000 are required to obtain a *NRMSE* of 2 % or less.

In averaging over several horizontal points to compare to a mean profile as is done above, some of the random noise and the microstructural features are averaged out or different microstructural features are measured when different horizontal points are sampled. Therefore, the Poisson estimate does not reliably predict the magnitude of the uncertainty in the horizontal averaging procedure. For the paste, the *NRMSE*s for all horizontal resolutions are lower than the Poisson estimate (Fig. 9), while for the mortar, some *NRMSE*s are higher by up to a factor of five and some are lower by up to a factor of four than the Poisson estimate (Fig. 10).

For the paste specimen, the Poisson estimate provides a reasonable approximation to the change in the uncertainty as the number of repetitions or the horizontal resolution changes. For example, at intensity *A* with one scan, the *NRMSE* is 4.5 % at a resolution of 10 mm (Fig. 9a). At a horizontal resolution of 5 mm, the *NRMSE* decreases to 2.8 %, or a factor of 1.6 smaller than that at a resolution of 10 mm. The Poisson estimate of this factor would be 1.4 since the number of counts increased by a factor of two in going from the 10 mm resolution to the 5 mm resolution.

For the mortar specimen, the Poisson estimate provides a reasonable estimate to the change in the uncertainty only for changes in horizontal resolution (Fig. 10). As mentioned above, the increased variability due to the presence of the aggregate masks any Poisson effect when averaging over several repetitions at a given horizontal resolution.

#### 3.3.2 Influence of the Number of Scanning Repetitions (Experiment *RVP*)

The paste is more sensitive to the number of scanning repetitions that are averaged together (Fig. 9) than is the mortar (Fig. 10). At intensity A with a horizontal resolution of 2 mm, the *NMRSE* for the paste is 2.2 % for one repetition and 1.2 % for four repetitions (Fig. 9a). Under these same conditions, the *NRMSE*s for the mortar are 2.0 % and 1.9 % (Fig. 10a). The larger small-scale variability due to the aggregates and the uncertainty in the positioning of the x-ray beam/detector combine to maintain a high *NRMSE* for the mortar even after averaging over four scans.

For both the paste and mortar, the dependence of the *NRMSE* on the number of repetitions decreases as the x-ray intensity increases (Figs. 9 and 10). For example, the range of *NRMSE* for the paste at intensity *C* with a 2 mm horizontal resolution is 0.3 % to 0.25 % from one to four scans (Fig. 9c), compared to 2.2 % to 1.2 % at intensity *A* (Fig. 9a). By increasing the x-ray intensity, more accurate data are obtained with fewer scans.

#### 3.3.3 Influence of X-Ray Source Intensity (Experiment *IVP* With Paste and Mortar)

The *NRMSE*s of the paste vertical profiles decreased with increasing intensity (Fig. 9). For example, at a horizontal resolution of 1 mm for intensity *A* and one scan, the *NRMSE* is about 1.7 % while that for intensity *C* is 0.3 % (Figs. 9a and 9c). For mortars, the *NRMSE* also decreases with increasing intensity from 0.8 % to 0.3 % from intensity *A* to *C* for one scan (Figs. 10a and 10c). These results suggest that higher x-ray intensities result in lower uncertainties, as seen above. Note that *NRMSE*s for all intensities, for just one scan, and for horizontal spacings of 2 mm or less are <2.2 % for both the paste and mortar.

The mortar scans produced counts that are roughly twice those for the pastes (Figs. 9 and 10). The measured density of the mortar is ≈2250 kg m^–3^ and the measured density of the paste is ≈1900 kg m^–3^. Therefore, the higher counts from the mortar specimen must be the result of changes in the composition of the material in comparison to the paste. In making the mortar, some cement was replaced by silica aggregate. By increasing the silica content and by reducing the calcium silicate content in the specimen by removing cement, the total absorption/scattering cross-section, e.g., Ref. [18] and the mass extinction coefficient of the specimen (Eq. 1) decreased. Higher counts for the mortar resulted.

### 3.4 Detecting Edges in a Specimen (Experiment *WEVP*: an Epoxy Block in Water)

**Fig. 11:**
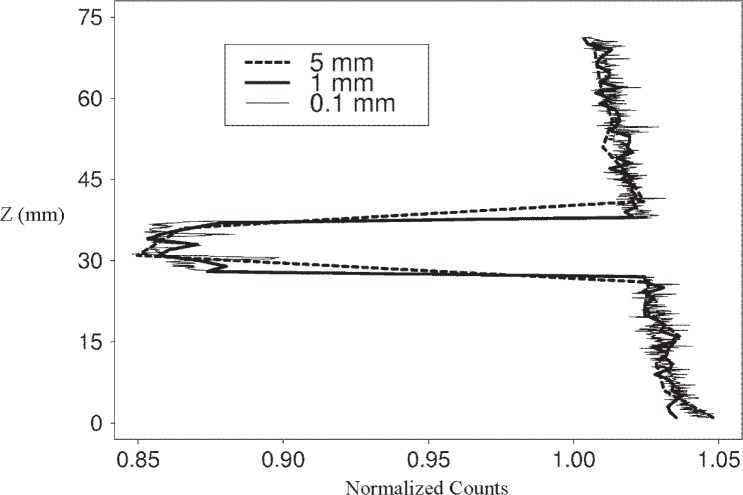
Normalized Counts from the *WEVP* experiments in which a water-filled cuvette with an epoxy block placed inside the cuvette near its middle was sampled.

Experiment *WEVP* was designed to determine how well the x-ray absorption system is able to detect the edges of an object. For this experiment, a cube of epoxy was placed in a water-filled smaller plastic cuvette and scanned at vertical resolutions of (0.1, 0.5, 1, 2.5, and 5) mm (Fig. 11). The true count profile for this specimen is given by the scan at a 0.1 mm vertical resolution. The epoxy block has lower counts than the water. The negative slope of the water portions of the profiles is the result of the cuvette being 0.7 mm wider at the top than it is at the bottom. In other words, the x-ray beam was more attenuated near the top of the specimen due to a longer path length. Note that all of the counts in the water regions are greater than one because the average counts over the entire profile was used to normalize the individual counts.

**Fig. 12:**
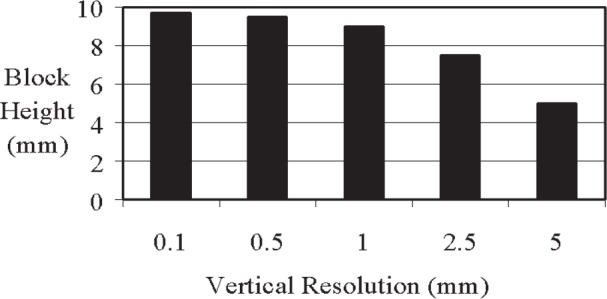
Estimates of the epoxy block height based on the count data from experiment *WEVP*. The actual height of the block is 10 mm.

The actual height of the block is 10 mm. Those locations whose normalized counts are 1.01 or lower were considered to be part of the block. This value was chosen because it represents the lowest normalized count that might be expected from the water portions of the profile in the area near the block based on the 0.1 mm scan. From this procedure, the estimates for the block height range from 5 mm for a vertical resolution of 5 mm to 9.7 mm for a vertical resolution of 0.1 mm (Fig. 12). With a vertical resolution of 0.5 mm or 1.0 mm, the machine estimate of the block height is 9 mm or higher. As an upper bound to the estimate of the block size, a linear interpolation to the normalized count level of 1.01 was done for the 5 mm scan. At this position, the estimate of the block height is 13 mm. This procedure may be useful in approximating the size of objects in a specimen when lower resolution scans are performed. Note that the scan for the 0.1 mm vertical resolution shows two relative maxima in normalized counts within the block area that are not seen in the other scans. These maxima correspond to air bubbles present in the epoxy cube.

The x-ray absorption system when used with a high vertical resolution (1 mm spacing or smaller–a factor of 10 or more smaller than the block) provides a representation of the vertical dimension of the epoxy block within 10 % of the actual dimension. The resolution chosen for use should be consistent with the level of detail that is desired for the object being scanned.

## 4. Summary and Discussion

X-ray absorption measurements are becoming more common in tracking moisture content and movement in materials such as cement pastes, mortars, and wood. However, no published data describing in detail the uncertainty and accuracy in the measurements due to various machine settings and different specimen materials were found. In this paper, the results of several experiments that were conducted to elucidate the uncertainty of measurements from an x-ray absorption machine and to determine the proper averaging procedures to reduce random noise in the data were presented.

X-ray absorption measurements are often thought of as a Poisson process in which the uncertainty of the measurements is the square root of the number of x-ray counts at a given point [6,7]. Based on the tests performed within, this estimate of uncertainty is most accurate in magnitude and trend when considering one point or several points of a uniform specimen like water. Experiments with water showed the uncertainty as indicated by the normalized root mean square error (*NRMSE*) to be within 25 % to 60 % of the Poisson estimate. The Poisson estimates and the *NRMSE*s agreed that when the number of counts increases, the uncertainty decreases, whether the increase in counts is due to increasing the x-ray intensity, the collimator size, or the integration time.

When averaging data points together, some of the random noise, the physical microstructure, or machine error due to positioning is averaged out. A comparison of *NRMSE*s and normalized standard deviations (*NSD*) was shown to have promise in determining if the profile structure that is measured is a real physical structure or the result of machine errors. More tests are needed to determine the utility of such comparisons.

For those cases in which random noise was averaged out, such as when the same vertical profile is measured several times, averaging up to four profiles together at each point can reduce the uncertainty by up to a factor of two. In this situation, the Poisson estimate may be considered the maximum uncertainty. When averaging several profiles from different locations in a specimen, some microstructural features may be added to or averaged out of the mean profile. The Poisson estimate in this case is no longer reliable in predicting the magnitude of the uncertainty, but may provide a good estimate of the change in uncertainty due to different averaging procedure. The *NRMSE*s for a paste specimen for which several vertical profiles of counts at different horizontal locations were averaged together were generally less than the Poisson estimate by as much as a factor of two. For a mortar specimen, some *NRMSE*s were higher by up to a factor of five and some were lower by up to a factor of four.

Such spatial averaging should only be performed in cases where it is conclusively known that the boundaries are not altering the microstructure in the direction being averaged. For example, averaging several points in a horizontal line to get one point of a vertical profile is only valid when the boundaries at either end of the horizontal line do not induce an effect on density or composition along that line.

The *NRMSE* was less than 2 % for a vertical profile of a uniform water specimen and was about 1 % when the same point was sampled continuously for counts exceeding 17 000. The expected uncertainty in approximating the mean profile of a cement paste specimen may range from less than 1 % to almost 5 % depending on the horizontal resolution and the number of scans that are averaged. For a mortar specimen, this range may be from less than 1 % to almost 10 %. To keep the *NRMSE*s below 2 % for the mortar specimen examined here, it was necessary to scan a sample at a horizontal resolution of 2 mm or less with counts of 4000 or higher. For the paste specimen studied here, a horizontal resolution of 10 mm was sufficient to obtain a *NRMSE* of 2 % or less with counts of 1500 or higher. For the paste and mortar specimens, when counts were above 5000, one scan of the specimen at a given horizontal resolution provided data within one percentage point of the average of up to four scans. With counts per second of greater than 1000, an integration time of 5 s produced uncertainties of 2 % or less.

Because the uncertainties change depending on the settings that are chosen and depending on the composition and structure of the specimen being scanned, it is impossible to determine ideal settings and averaging procedures that will be valid in every circumstance. The values summarized above are intended as a guide. For more specific information about scans of a given specimen, it is recommended first that the degree of accuracy or uncertainty that is desired be determined. Next, a specimen of a uniform material such as aluminum or water of the same size as the actual specimen to be scanned should be used with various machine settings to get an idea of the base uncertainty for a specimen this size with these possible settings. After this, at least one specimen should be scanned at a very high horizontal and vertical resolution to determine the true structure of the specimen. Then, lower resolution scans can be checked against the more detailed one to see how well they match. By repeating this procedure, the uncertainties of the measurements and the proper machine settings and averaging procedures can be determined.

Finally, the process of x-ray absorption is not only dependent on the density and thickness of specimen but also on its composition. This work showed that while a mortar specimen was about 20 % denser than a paste specimen, the mortar counts were twice as large as those for the pastes. This fact must be remembered when comparing counts from two specimens of differing composition.

## Figures and Tables

**Table 1.** Description of the experiments used to examine the abilities of the x-ray absorption system. The “Variable” row gives the machine or sampling parameter varied. “Inherent variability” refers to the overall uncertainty related to the chosen machine settings

Experiment	CVP	IVP	OP	RVP	WEVP	WVP	XVP
Variable	Collimator size	X-ray intensity	Inherent variability	Scanning repetitions	Vertical resolution	Inherent Variability/Integration time	Horizontal resolution
Data structure	Vertical count profiles	Vertical count profiles	Counts at one point	Vertical count profiles	Vertical count profiles	Vertical count profiles	Vertical count profiles
Specimen dimensions (mm)	20 × 20 × 90	20 × 20 × 90	20 × 55 × 60	20 × 20 × 90	10 × 10 × 40	20 × 55 × 60	20 × 20 × 90
Specimen holder material	Acrylic	Acrylic	Plastic	Acrylic	Plastic	Plastic	Acrylic
Specimen material	Paste/mortar/water	Paste/mortar/water	Water	Paste/mortar/	Water/	Water epoxy	Paste/mortar/
Voltage/current		A: 38 kV/300 µA	A: 27 kV/200 µA	A: 38 kV/300 µA		A: 27 kV/200 µA	A: 38 kV/300 µA
	43 kV/700 µA	B: 40 kV 500 µA	B: 35 kV 200 µA	B: 40 kV 500 µA	35 kV 300 µA	B: 35 kV 200 µA	B: 40 kV 500 µA
		C: 43 kV 700 µA	C: 45 kV 200 µA	C: 43 kV 700 µA		C: 45 kV 200 µA	C: 43 kV 700 µA
Integration time (s)	5	5	5	5	1, 5, 10, 20, 30	5	5
Number of scanning repetitions	1	1, 2, 3, 4	100	1, 2, 3, 4	6	4	1, 2, 3, 4
Vertical scanning resolution (mm)	1	1		1	0.1, 0.5, 1, 2.5, 5	1	1
Horizontal scanning resolution (mm)	1, 2, 5, 10			1, 2, 5, 10			1, 2, 5, 10
Collimator number (see Table 2)	All	5	5	5	5	5	5

**Table 2:** Information about the collimators used in the x-ray absorption system. The “Number” column refers to the collimator number. D in the “Shape and dimensions” column represents the diameter of the circular openings of the collimators. The “Area factor” gives the factor that when multiplied by the number of counts from the corresponding collimator would estimate the number of counts for the square collimator (collimator #2) at the same x-ray intensity. The “Water counts,” “Paste counts,” and “Mortar counts” refer to the CVP experiments (see Table 1)

Number	Shape and dimensions (mm)	Area factor	Paste counts	Paste area factor	Mortar counts	Mortar area factor	Water counts	Water area factor
1	Horizontal Ellipsoid (0.5 × 0.1)	229.0	2144	130.0	4396	123.3	5930	124.6
2	Square (3 × 3)	1	278 009	1	542 105	1	739 036	1
3	Circle (*D* = 2)	2.9	82 562	3.4	166 448	3.3	227 821	3.2
4	Circle (*D* = 1)	11.5	26 788	10.4	54 299	10.0	74 542	9.9
5	Circle (*D* = 5)	5.9	6053	45.9	12 152	44.6	16 758	44.1
6	Circle (*D* = 0.16)	450.0	700	397.2	1315	412.2	1723	428.9
7	Vertical ellipsoid (0.1 × 0.5)	229.0	1341	207.3	2577	210.4	3500	211.1

**Table 3:** Sieve size analysis for the aggregates used in the mortars. The four aggregates used were U.S. Silica Company’s F95 and S15 and ASTM Graded Sand and 20-30 sand. See Snyder et al. [16] for more information about this aggregate mix. The “Dimension” column gives the length of the square openings on the sieves

Sieve size #	Dimension (mm)	Percent passing
8.0	2.360	100.0
10.0	2.000	99.4
12.0	1.700	93.9
16.0	1.180	66.1
20.0	0.850	60.7
30.0	0.600	44.8
40.0	0.425	38.3
50.0	0.300	29.5
70.0	0.212	24.7
100.0	0.150	15.1
140.0	0.106	4.8
200.0	0.075	0.8

**Table 4:** Statistics from the water experiment *OP* in which the same point was sampled 100 times at three different intensities. *NSD* represents the normalized standard deviation. *NRMSE* is the normalized root mean square error with the measurement at intensity *C* used as the true value. *N* is the average number of counts per point and *N* ^–0.5^ represents the uncertainty estimate from the Poisson approach. See Table 1 for more information on experiment *OP*

		Intensity		
		*A* 27 kV; 200 µA	*B* 35 kV; 200 µA	*C* 45 kV; 200 µA
Water	*NRMSE*	0.035	0.012	
	*NSD*	0.036	0.011	0.005
	*N* ^–0.5^	0.027	0.008	0.003
	*N*	1378	16 905	91 039

**Table 5:** Uncertainty estimates and the average number of counts per point for four vertical profiles at the same horizontal location for three x-ray source intensities and for water, paste, and mortar specimens (experiment *IVP*). The normalized root mean square error (*NRMSE*) is presented two ways. First, it is computed as the average of the four *NRMSE*s at each intensity in comparison to the mean profile at the highest intensity, *C* (*NRMSE*a). Second, *NRMSE*b is computed as the *NRMSE* of the mean profile for each intensity in comparison to the mean profile at intensity *C*. The normalized standard deviation (*NSD*) is presented both as the average of the four *NSD*s (*NSD*a) and as the *NSD* of the mean profile (*NSD*b). *N* represents the average number of counts per point in the profiles. *N* ^–0.5^ represents the uncertainty estimates from the Poisson approach. See Table 1 for more information on experiment *IVP*

		Intensity		
		*A* 38 kV; 300 µA	*B* 40 kV; 500 µA	*C* 43 kV; 700 µA
Water	*NRMSE*a	0.036	0.012	0.004
	*NRMSE*b	0.017	0.006	
	*NSD*a	0.036	0.012	0.005
	*NSD*b	0.017	0.005	0.002
	*N* ^–0.5^	0.027	0.008	0.003
	*N*	1395	16 492	91 221
Paste	*NRMSE*a	0.070	0.034	0.015
	*NRMSE*b	0.039	0.018	
	*NSD*a	0.098	0.077	0.073
	*NSD*b	0.079	0.071	0.072
	*N* ^–0.5^	0.044	0.025	0.013
	*N*	528	1661	6149
Mortar	*NRMSE*a	0.048	0.037	0.013
	*NRMSE*b	0.032	0.030	
	*NSD*a	0.100	0.095	0.083
	*NSD*b	0.098	0.092	0.082
	*N* ^–0.5^	0.029	0.016	0.009
	*N*	1223	3745	12 462

## Footnotes

1 Certain commercial equipment, instruments, or materials are identified in this paper to foster understanding. Such identification does not imply recommendation or endorsement by the National Institute of Standards and Technology, nor does it imply that the materials or equipment identified are necessarily the best available for the purpose.
